# When Wholes Resist Decomposition: A Spectral Measure of Epistemic Emergence

**DOI:** 10.3390/e28040380

**Published:** 2026-03-28

**Authors:** Mark Bailey, Susan Schneider

**Affiliations:** 1 Department of AI, Cyber, Influence and Data Science, National Intelligence University, Bethesda, MD 20816, USA; 2 Biological and Computational Intelligence Center, National Intelligence University, Bethesda, MD 20816, USA; 3 Center for the Future Mind, AI & Society, Florida Atlantic University, Boca Raton, FL 33431, USA; 4 School of Arts and Letters, Florida Atlantic University, Boca Raton, FL 33431, USA

**Keywords:** integrated information, weak IIT, epistemic emergence, spectral graph theory, mutual information, complex systems, multi-agent systems, irreducibility

## Abstract

Multi-agent and distributed dynamical systems can exhibit coordinated behavior that is difficult to summarize in terms of independent parts. Integrated Information Theory (IIT) provides one influential notion of system-level irreducibility, but exact computation of causal Φ remains intractable except in very small systems. In this work, we introduce $\Phi_{\mathrm{spectral}}$, a scalable observer-relative statistic defined on pairwise mutual information networks extracted from multivariate time-series data. A normalized graph Laplacian and its Fiedler vector identify a bipartition of the mutual information graph, and $\Phi_{\mathrm{spectral}}$ reports the normalized weight of informational coupling crossing that cut. The measure is inspired by IIT’s concern with irreducibility but is not equivalent to intrinsic causal Φ: it is pairwise, undirected, and functional/statistical rather than intervention-based. We evaluate it on four exploratory simulation regimes: random oscillators, a transitional Kuramoto-like synchronization regime, a perfectly synchronized regime, and a combinatorial threshold-linear network (CTLN). Across these cases, $\Phi_{\mathrm{spectral}}$ is most useful as a measure of observer-relative integration under second-order dependencies, separating redundancy-dominated from transiently differentiated regimes. The current results should be read as a proof-of-concept rather than as a formal validation against exact IIT. We discuss relations to weak IIT, Integrated World Modeling Theory (IWMT), and the perturbational complexity index (PCI), and we outline the stationary benchmarking and small-system validation needed for stronger causal claims.

## 1. Introduction

Modern multi-agent systems—ranging from autonomous drone swarms [1,2] to hybrid human–machine teams [3,4] and large AI ecosystems [5,6,7]—increasingly exhibit behaviors that appear coordinated, adaptive, and intelligent at the group level. These systems often display emergent capabilities such as collective inference, distributed planning, and robust self-organization [8,9]. A central challenge is to quantify when such behavior reflects a merely coordinated collection of parts and when it reflects a system that, for practical purposes, resists decomposition.

Integrated Information Theory (IIT) offers one influential way of framing this question. In IIT, a system’s integrated information, denoted Φ, measures the degree to which its cause–effect structure is irreducible under partition [10,11,12,13]. This idea has motivated work not only on consciousness, where IIT originated, but also on causal emergence and complex systems more broadly [14,15,16]. However, exact computation of system-level Φ rapidly becomes infeasible because it requires comparing many mechanisms, purviews, and partitions [17,18].

This computational barrier has encouraged a broader search for empirically useful measures of integration, complexity, and irreducibility. Candidate approaches include synergy- and redundancy-oriented decompositions [19], multi-information and total correlation [20,21], practical time-series measures [22], information-geometric approaches [23], and perturbation-based indices such as PCI [24]. Recent work on weak IIT explicitly argues for separating the empirical study of information dynamics from the stronger metaphysical and intrinsic commitments of IIT’s full formalism [25].

The present paper contributes to that weaker, observer-relative program. We introduce $\Phi_{\mathrm{spectral}}$, a normalized spectral statistic computed from pairwise mutual information graphs estimated from multivariate time-series data. The Fiedler vector of the normalized Laplacian identifies a bipartition that approximately minimizes a normalized cut, and $\Phi_{\mathrm{spectral}}$ reports the fraction of total pairwise mutual information that crosses that partition. This makes the measure scalable and easy to compute from observed data alone. At the same time, it also places clear limits on interpretation: $\Phi_{\mathrm{spectral}}$ is not a validated estimator of IIT’s intrinsic causal Φ. It is pairwise rather than multivariate, undirected rather than causal, and observer-relative rather than intrinsic.

Our aim is to determine whether this spectral statistic behaves in an intelligible way across four exploratory dynamical regimes: uncoupled oscillators, a non-stationary transition toward synchrony, a perfectly synchronized redundant regime, and a combinatorial threshold-linear network (CTLN). These cases allow us to separate three ideas that are often conflated: coordination, redundancy, and observer-relative integration. We also clarify how the proposed statistic relates to weak IIT, IWMT, and PCI, and we identify the validation steps still required before stronger causal or consciousness-related claims would be warranted.

## 2. Theoretical Background

### 2.1. Emergence and Observer-Relative Irreducibility

The notion of emergence occupies a central place in both philosophy of science and the study of complex systems. At a high level, an emergent property is a macroscopic feature of a system that is not straightforwardly recoverable from an inspection of local parts in isolation. Philosophical discussions often distinguish between strong emergence, where the irreducibility is taken to hold in principle, and weak or epistemic emergence, where the difficulty is one of explanation, prediction, or compression for a bounded observer [26,27]. Related work also distinguishes synchronic emergence, concerning constitutive organization at a time, from diachronic emergence, concerning novelty that depends on temporal development [28,29].

The measure proposed here is best interpreted within this weaker, observer-relative setting. $\Phi_{\mathrm{spectral}}$ is computed from pairwise statistical dependencies accessible to an external observer; it therefore quantifies practical inseparability under a restricted descriptive vocabulary, not ontological novelty or intrinsic cause–effect power. A high value indicates that second-order informational dependencies are distributed across a system in a way that resists decomposition by a natural spectral partition. In this sense, the measure targets practical irreducibility rather than metaphysical emergence.

Operationally, the present implementation is even narrower than that philosophical framing may suggest. We estimate $\Phi_{\mathrm{spectral}}$ in sliding windows and examine the resulting time series descriptively. The statistic is therefore best understood as a synchronic-within-window quantity whose trajectory may suggest changing integration over time. It is not yet a standalone estimator of diachronic emergence in the stronger historical sense.

### 2.2. IIT, Weak IIT, and the Scope of the Present Measure

IIT provides a precise and ambitious account of irreducibility. In its contemporary formulations, a system has high integrated information when its cause–effect structure cannot be factorized without significant loss [12,30]. System-level Φ is defined by comparing the full cause–effect structure to that obtained under a minimum information partition, with the comparison grounded in interventional repertoires rather than passive observation [11,13]. This makes IIT a theory of intrinsic causal organization, not merely of correlation or compressibility.

That distinction matters here. The computational appeal of a spectral graph approach does not erase the conceptual difference between intrinsic causal structure and observer-relative functional dependence. Accordingly, we do not claim that $\Phi_{\mathrm{spectral}}$ is equal to, or formally validated as an approximation of, exact IIT Φ. Instead, the present measure should be understood as a scalable statistic inspired by IIT’s concern with irreducibility while remaining much closer to weak IIT and other empirical approximation programs [22,25,31].

This positioning also clarifies our stance on scale. Because $\Phi_{\mathrm{spectral}}$ can be computed for whole systems or for candidate subsystems, it does not presuppose IIT’s exclusion axiom or a unique maximally integrated complex. That multiscale flexibility is deliberate and aligns better with weak-IIT-style empirical work and with broader integrative frameworks such as IWMT, which treat information integration as one ingredient within a larger theory of modeling, embodiment, and agentic organization [25,32,33].

### 2.3. Definition of the Spectral Statistic

Let $X=\{X_{1},\ldots ,X_{n}\}$ denote *n* observed component time series within a given window, and let $M\in R^{n\times n}$ be the symmetric mutual information matrix with entries

$$M_{ij}=I\left(X_{i};X_{j}\right),\;M_{ii}=0.$$

Define the degree matrix *D* by $D_{ii}=\sum_{j}M_{ij}$, and form the normalized graph Laplacian

$$L=I-D^{-1/2}MD^{-1/2}.$$

Let $v_{2}$ denote the Fiedler vector, i.e., the eigenvector associated with the second-smallest eigenvalue of *L* [34,35]. The sign structure of $v_{2}$ induces a bipartition A∪B=V of the nodes. We then define the normalized spectral cut weight

$$\Phi_{\mathrm{spectral}}=\left\{\begin{array}{cc} \frac{\sum_{i\in A,\;j\in B}M_{ij}}{\sum_{1\leq i<j\leq n}M_{ij}}, & \mathrm{if}\;\sum_{1\leq i<j\leq n}M_{ij}>0,\\ 0, & \mathrm{otherwise}. \end{array}\right.$$

This normalized statistic takes values in [0,1] and is the quantity plotted in all figures below. Informally, it reports the fraction of total pairwise mutual information that must cross the Fiedler bipartition.

**Figure 1:**
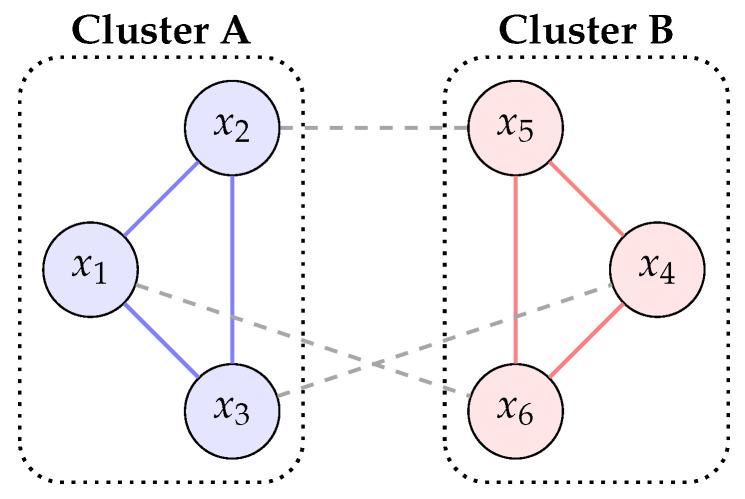
Schematic Fiedler bipartition of a mutual information graph. Solid edges indicate stronger within-cluster dependencies; dashed edges indicate the cross-cut dependencies summarized by $\Phi_{\mathrm{spectral}}$.

The rationale for this construction is heuristic rather than theorem-level. The Fiedler partition is widely used as a computationally efficient approximation to a normalized minimal cut. In the present setting, it yields a natural “least disruptive” bipartition of the pairwise mutual information graph. A high $\Phi_{\mathrm{spectral}}$ indicates that second-order dependencies remain broadly distributed across that cut; a low value indicates that most pairwise dependence can be localized within the two sides of the partition (Figure 1).

### 2.4. Mutual Information as Edge Weight and Resulting Limitations

Mutual information is a natural edge weight because it is symmetric, non-negative, and sensitive to nonlinear as well as linear dependencies. For discrete random variables $X_{i}$ and $X_{j}$,

$$I\left(X_{i};X_{j}\right)=\sum_{x_{i}}\sum_{x_{j}}p\left(x_{i},x_{j}\right)\log\frac{p(x_{i},x_{j})}{p\left(x_{i}\right)p\left(x_{j}\right)}=H\left(X_{i}\right)+H\left(X_{j}\right)-H\left(X_{i},X_{j}\right).$$

Using mutual information therefore allows us to build a weighted, undirected graph directly from observed data without assuming a parametric dynamical model.

At the same time, this choice sharply delimits what the measure can mean. Mutual information is not directional, is not interventional, and cannot by itself separate redundant from synergistic higher-order structure. Moreover, in a windowed setting, its empirical estimate depends on binning, window length, and the extent to which the process is approximately stationary within each window. These caveats apply directly to the present experiments.

Accordingly, $\Phi_{\mathrm{spectral}}$ should be interpreted as an observer-relative statistic of second-order functional integration. It is useful precisely because it is tractable and descriptive. It should not be mistaken for a full account of intrinsic causal irreducibility.

## 3. Materials and Methods

### 3.1. Overview

To illustrate the behavior of $\Phi_{\mathrm{spectral}}$, we analyze four simulated systems with different dependency structures: (1) uncoupled random oscillators, (2) a non-stationary transition toward synchrony, (3) a perfectly synchronized redundant regime, and (4) a combinatorial threshold-linear network (CTLN) with graph-constrained dynamics. The first three conditions are best understood as Kuramoto-like phase-oscillator baselines rather than as realistic models of causal interaction [36]. Our goal is exploratory: to test whether the statistic distinguishes independence, gradual synchronization, total redundancy, and topology-driven structured dynamics.

### 3.2. System Configuration and Agent Dynamics

We consider a system of *n* agents evolving over *T* discrete time steps. Each agent *i* has a scalar state $x_{i}^{t}\in \left[-1,1\right]$ at time *t*, corresponding either to the sine of a phase variable $\theta_{i}\left(t\right)$ or, in the CTLN case, to a threshold-linear activity variable.

**Random oscillators.**

In the uncoupled baseline, each oscillator evolves independently as

$$x_{i}^{t}=\sin\left(2\pi f_{i}t+\phi_{i}\right),$$

where $f_{i}∼U\left(f_{\min},f_{\max}\right)$ and $\phi_{i}∼U\left(0,2\pi \right)$. This condition contains no designed coupling and therefore serves as a control for finite-window and discretization effects.

**Transitional oscillators.**

In the transitional condition, oscillators begin incoherent but are progressively coupled through a mean-field term,

$$\theta_{i}^{t+1}=\theta_{i}^{t}+\omega_{i}+K\left(t\right)\sin\left({\bar{\theta }}^{t}-\theta_{i}^{t}\right),$$

where $\omega_{i}$ is the intrinsic frequency, ${\bar{\theta }}^{t}$ is the mean phase, and K(t) increases sigmoidally over time. The observed state is $x_{i}^{t}=\sin\left(\theta_{i}^{t}\right)$. Because K(t) is explicitly time-varying, this condition is non-stationary by construction and is included as an exploratory example rather than as a stationary benchmark.

**Synchronized oscillators.**

In the synchronized condition, all oscillators share the same phase and frequency,

$$x_{i}^{t}=\sin\left(2\pi ft\right),\;\forall i.$$

This produces a maximally redundant regime. In the limit of a uniform mutual information graph, the Fiedler eigenspace is degenerate, so this condition is also useful for exposing when the spectral partition becomes poorly informative.

**Combinatorial threshold-linear network (CTLN).**

For a more structured test case, we examine a CTLN, following Curto and collaborators [37]. CTLNs are recurrent neural systems whose dynamics are determined by a directed graph G=(V,E). Node activity evolves as

$$\frac{dx_{i}}{dt}=-x_{i}+{\left[\sum_{j=1}^{n}W_{ij}x_{j}+\theta \right]}_{+},$$

where ${\left[\cdot \right]}_{+}=\max\left\{0,\cdot \right\}$, θ>0 is the external drive, and the connection matrix is

$$W_{ij}=\left\{\begin{array}{cc} 0 & \mathrm{if}\;i=j,\\ -1+\varepsilon & \mathrm{if}\;j\to i\in E,\\ -1-\delta & \mathrm{if}\;j\to i\notin E. \end{array}\right.$$

Here, ε∈(0,1) and δ>0 determine weak and strong inhibition, respectively. CTLNs provide a convenient example in which the interaction graph shapes transient and attractor structure directly.

### 3.3. Mutual Information Estimation

Given a time-series matrix $X\in R^{n\times T}$, we compute a mutual information matrix $M\in R^{n\times n}$ over sliding windows of width *w* and stride *s*. Within each window, each time series is discretized into *b* bins using uniform binning, and pairwise mutual information is estimated from empirical frequencies.

The present figures use short windows (w=10) and coarse discretization (b=3). These settings should be regarded as exploratory. In particular, short windows and coarse binning can create a nonzero estimation floor even for uncoupled systems, and they can amplify partition instability when the mutual information graph becomes nearly uniform.

### 3.4. Spectral Statistic over Time

For each window, we form the weighted mutual information graph, compute the normalized Laplacian, extract the Fiedler vector, and evaluate the normalized cut statistic

$$\Phi_{\mathrm{spectral}}\left(t\right)=\frac{\sum_{i\in A_{t},\;j\in B_{t}}M_{ij}^{\left(t\right)}}{\sum_{1\leq i<j\leq n}M_{ij}^{\left(t\right)}},$$

where $\left(A_{t},B_{t}\right)$ is the Fiedler bipartition for window *t*. This produces a bounded time series $\Phi_{\mathrm{spectral}}\left(t\right)\in \left[0,1\right]$.

Under this normalization, $\Phi_{\mathrm{spectral}}$ should be read as the proportion of total pairwise mutual information that remains distributed across the least-favorable spectral bipartition. In nearly uniform graphs, however, many cuts become near-equivalent; in that regime, the statistic can reflect partition balance as much as substantive dynamical change.

### 3.5. Implementation and Reproducibility

All simulations were implemented in Python v. 3.9 using NumPy v. 1.23, SciPy v. 1.1, scikit-learn v. 1.3, NetworkX v. 3.1, and matplotlib v. 3.7. CTLN dynamics were integrated using Euler’s method with Δt=0.1. Default parameters were n=50 agents, T=150 time steps, window size w=10, stride s=1, and b=3 bins. CTLN parameters were fixed at θ=1.0, ε=0.25, and δ=0.5 [37]. The traces shown below are single illustrative runs and should therefore be interpreted qualitatively. Future work should consider stationary parameter sweeps, multiple random seeds, and sensitivity analyses over *w*, *s*, and *b*.

## 4. Results

The four conditions illustrate both the usefulness and the current limits of $\Phi_{\mathrm{spectral}}$. The random baseline reveals the estimator’s floor under the chosen settings. The transitional and synchronized oscillator regimes show what happens when pairwise dependence becomes increasingly uniform and redundant. The CTLN regime provides a more structured case in which dependencies remain nonuniform over time.

### 4.1. Random Oscillators

**Figure 2:**
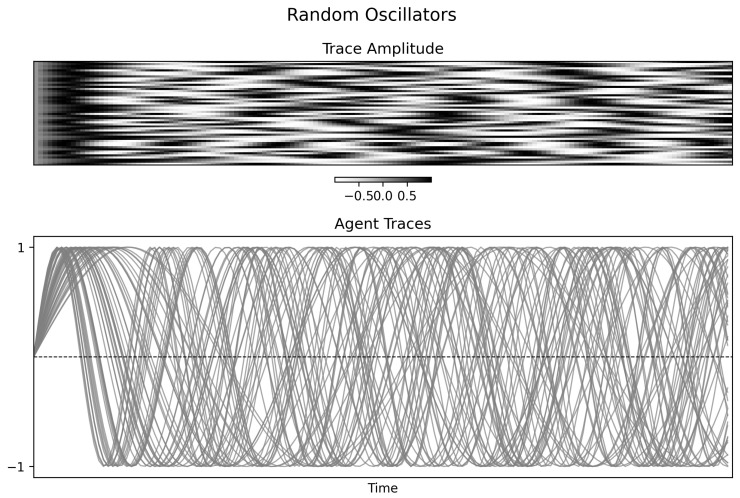
Time-series visualization of random oscillators. **Top panel**: Trace amplitudes over time, showing heterogeneous and uncoordinated activity. **Bottom panel**: Individual traces with differing phases and frequencies, with no persistent collective alignment.

**Figure 3:**
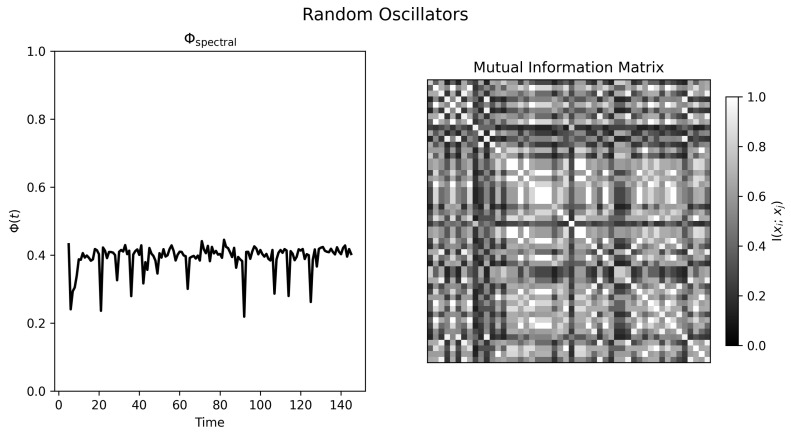
Normalized spectral statistic and mutual information structure for random oscillators. **Left panel**: $\Phi_{\mathrm{spectral}}\left(t\right)$ remains moderate and noisy, indicating a nonzero estimation floor under the current windowing and binning choices. **Right panel**: Mutual information matrix from the final time window, showing weak and irregular pairwise dependencies rather than stable organization.

In the uncoupled random condition, the agent traces show no stable collective organization (Figure 2). The corresponding $\Phi_{\mathrm{spectral}}\left(t\right)$ trace (Figure 3, left) fluctuates around a moderate baseline rather than collapsing to zero. Because the oscillators are uncoupled by design, that baseline should not be interpreted as genuine system-level integration. Instead, it reflects the combined effects of finite windows, coarse discretization, and accidental short-window phase alignments among simple periodic signals. The mutual information matrix (Figure 3, right) is correspondingly noisy and weakly structured.

### 4.2. Transitional Oscillators

**Figure 4:**
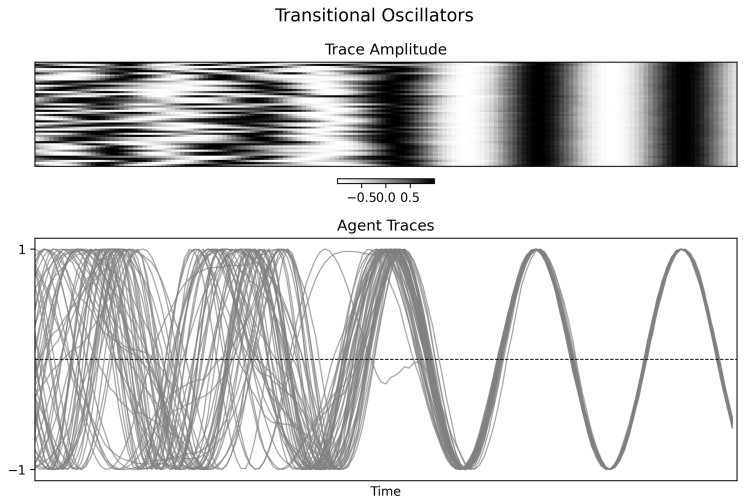
Time-series visualization of transitional oscillators. **Top panel**: Trace amplitudes over time, with increased alignment in the latter portion of the simulation. **Bottom panel**: Individual traces showing an initially heterogeneous regime followed by growing phase locking.

**Figure 5:**
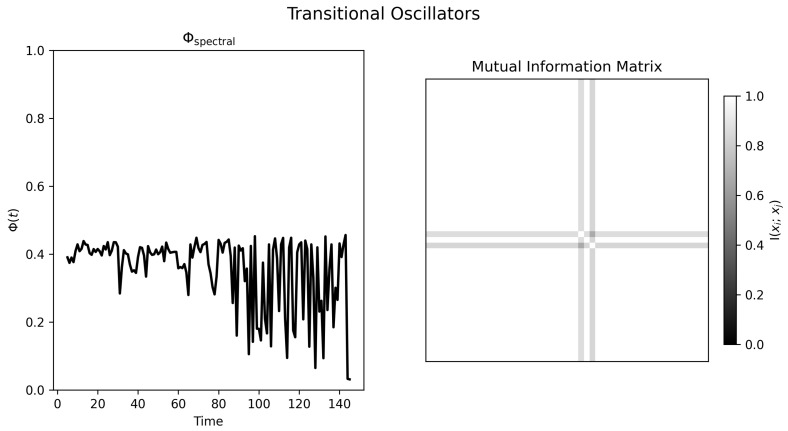
Normalized spectral statistic and mutual information structure for transitional oscillators. **Left panel**: $\Phi_{\mathrm{spectral}}\left(t\right)$ becomes more volatile late in the run rather than changing monotonically; in this regime, part of the variability likely reflects instability of the spectral cut in an increasingly redundant graph. **Right panel**: Mutual information matrix from the final time window, showing widespread near-uniform dependence consistent with late-stage redundancy.

The transitional condition is the most challenging to interpret because coupling changes explicitly with time. Figure 4 shows a progression from incoherence toward phase alignment. The final mutual information matrix (Figure 5, right) is nearly uniform, consistent with a highly redundant late-stage regime. However, the normalized $\Phi_{\mathrm{spectral}}\left(t\right)$ trace does not increase monotonically; instead it becomes increasingly volatile late in the run.

Under the present definition, this behavior is unsurprising. Once the mutual information graph approaches a nearly complete, nearly uniform graph, the Fiedler partition becomes weakly constrained and the statistic partly reflects changes in partition balance. Thus, the existing figure should be read as evidence of a transition from heterogeneous dependence toward redundancy.

### 4.3. Synchronized Oscillators

**Figure 6:**
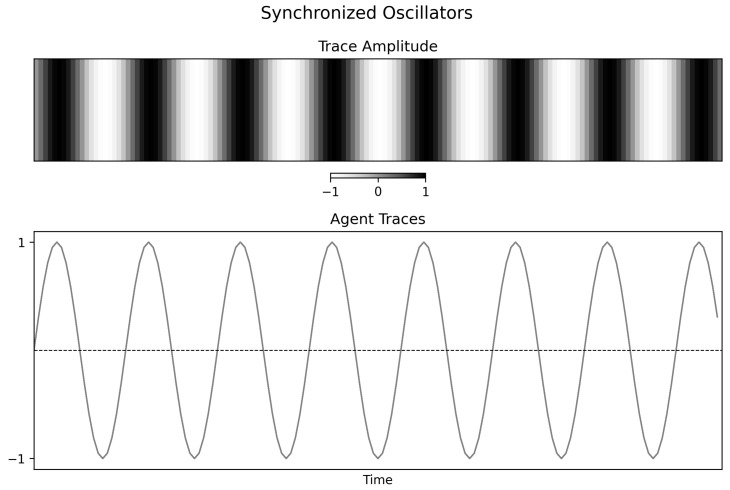
Time-series visualization of fully synchronized oscillators. **Top panel**: Trace amplitudes showing complete temporal alignment. **Bottom panel**: Indistinguishable traces in full phase and frequency lock.

**Figure 7:**
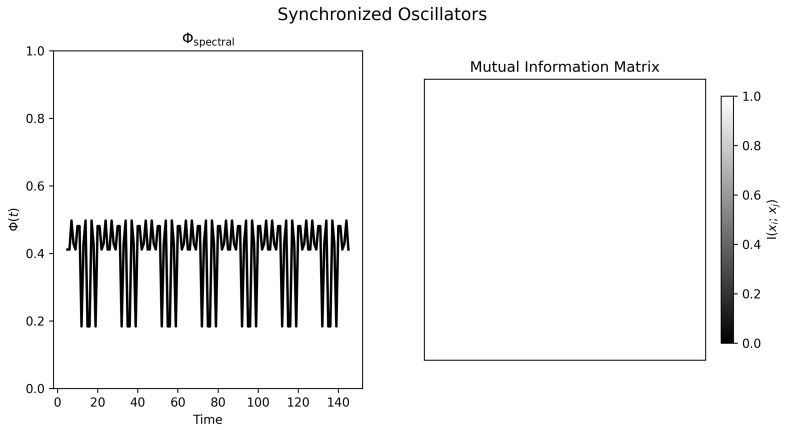
Normalized spectral statistic and mutual information structure for synchronized oscillators. **Left panel**: Variation in $\Phi_{\mathrm{spectral}}\left(t\right)$ largely reflects degeneracy of the spectral partition in a nearly uniform graph rather than substantive changes in the dynamics. **Right panel**: Final mutual information matrix showing near-uniform maximal pairwise dependence, consistent with extreme redundancy.

The synchronized condition makes the interpretive issue especially clear. All oscillators carry the same signal (Figure 6), and the final mutual information matrix is essentially saturated (Figure 7, right). In that limit, many bipartitions are nearly equivalent. The observed oscillations in $\Phi_{\mathrm{spectral}}\left(t\right)$ therefore should not be read as meaningful changes in interaction strength; they are best understood as consequences of partition degeneracy in an almost uniform graph.

The substantive lesson of this condition is therefore a negative one. Perfect synchrony does not produce a uniquely high value of this pairwise spectral statistic. Instead, total redundancy makes the statistic weakly informative because differentiation has collapsed.

### 4.4. Combinatorial Threshold-Linear Network (CTLN)

**Figure 8:**
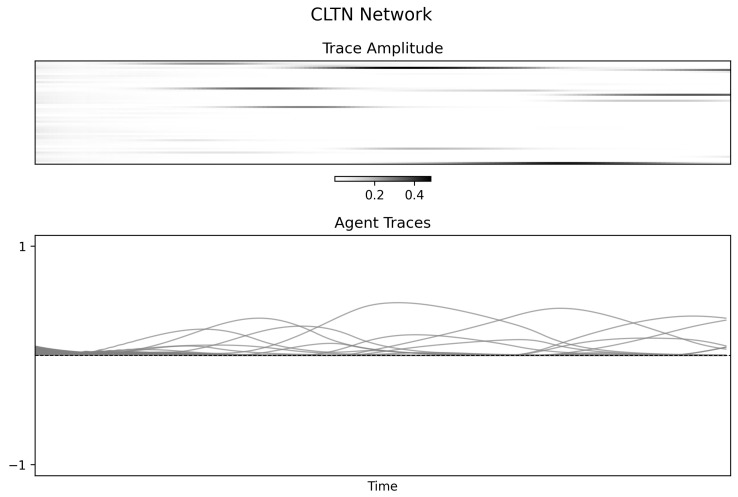
Time-series visualization of a combinatorial threshold-linear network (CTLN). **Top panel**: Trace amplitudes showing an initial transient followed by a sparse long-run regime. **Bottom panel**: Individual traces revealing early competition and subsequent convergence toward a lower-dimensional attractor.

**Figure 9:**
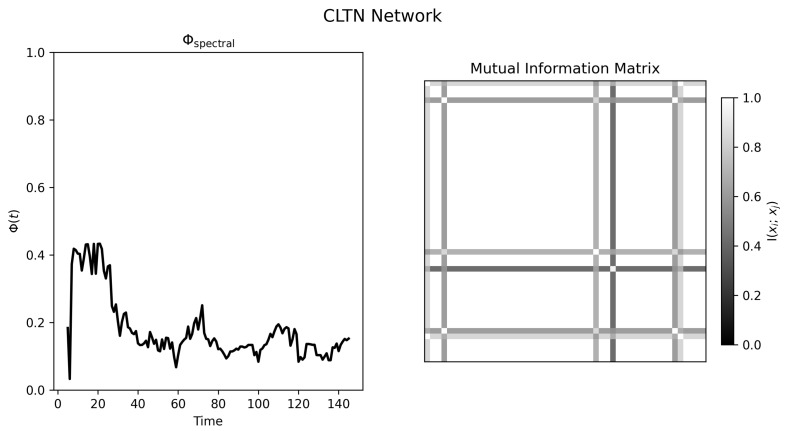
Normalized spectral statistic and mutual information structure for the CTLN simulation. **Left panel**: $\Phi_{\mathrm{spectral}}\left(t\right)$ declines after an early transient, consistent with compression into a sparse attractor. **Right panel**: Mutual information matrix from the final time window, showing structured but nonuniform dependencies concentrated in a subset of nodes.

The CTLN condition provides a more structured and informative example. Figure 8 shows an early transient with broader distributed activity followed by convergence toward a sparse attractor dominated by a subset of nodes. The $\Phi_{\mathrm{spectral}}\left(t\right)$ trace (Figure 9, left) declines correspondingly. Unlike the synchronized oscillator regime, the final mutual information matrix remains structured but nonuniform, with dependencies concentrated around a subset of units rather than distributed uniformly across the entire graph.

This pattern is consistent with a system whose interactions compress over time into a lower-dimensional regime. Because the CTLN trajectory is also non-stationary during the transient, the result should still be interpreted qualitatively. Even so, this condition provides the clearest demonstration in the present paper that $\Phi_{\mathrm{spectral}}$ can track structural simplification in a graph-constrained dynamical system.

### 4.5. Comparative Analysis

Taken together, the figures support three conclusions. First, $\Phi_{\mathrm{spectral}}$ distinguishes noisy independence, near-uniform redundancy, and topology-driven structured compression. Second, in nearly uniform graphs—especially under full synchrony—the statistic becomes sensitive to Fiedler-cut degeneracy and should be interpreted cautiously. Third, structured network dynamics such as those of the CTLN provide more informative test beds for the method than pure oscillator baselines.

What the current figures do not establish is equally important. They do not validate $\Phi_{\mathrm{spectral}}$ as an estimator of exact IIT Φ, and they do not yet provide a stationary characterization of how the statistic changes with coupling strength. Those stronger claims require additional simulations and explicit benchmarking against tractable small systems.

## 5. Discussion

The main contribution of this work is methodological rather than doctrinal. We have defined a scalable statistic of observer-relative integration on pairwise mutual information graphs and shown, using the present exploratory simulations, where it behaves intuitively and where it does not. The resulting picture is useful precisely because it is limited: $\Phi_{\mathrm{spectral}}$ is a descriptive index of how second-order dependencies distribute across a natural spectral bipartition, not a surrogate for IIT’s intrinsic causal Φ.

**Relation to weak IIT.** This interpretation places the proposal closer to weak IIT than to strong IIT. Weak IIT treats information-dynamic measures as empirically useful hypotheses about consciousness and integration without requiring that any one measure fully instantiate the formal and metaphysical commitments of IIT [25]. Within that broader program, $\Phi_{\mathrm{spectral}}$ is best viewed as a tractable screen for observer-relative integration in systems too large or poorly understood for exact causal modeling.

**What the oscillator baselines reveal.** The oscillator examples are revealing mainly because they expose two limitations clearly. The random baseline shows that short windows and coarse binning induce a nonzero floor. The transitional and synchronized conditions show that once pairwise dependence becomes nearly uniform, the spectral cut loses specificity. This is a generic consequence of summarizing an almost complete uniform graph by a single bipartition.

**Why the CTLN case is more informative.** The CTLN regime is therefore especially useful. Here the dependency structure remains nonuniform and graph-shaped even as the system compresses into a sparse attractor. In this setting, the statistic tracks structural simplification more coherently. That is close to the application for which the measure seems most promising: monitoring the rise or collapse of distributed, but not fully homogeneous, organization.

**Connections to IWMT, harmonic modes, and expander-style intuitions.** IWMT offers a suggestive point of contact because it discusses integrated complexes in terms of harmonic or oscillatory modes over connectivity structure and explores how Laplacian-like descriptions, turbo-coding, shared latent spaces, and graph-theoretic architectures may support world modeling and consciousness [32,33]. Our use of the graph Laplacian resonates with that literature formally, but the overlap should not be overstated. $\Phi_{\mathrm{spectral}}$ is built from passive pairwise mutual information on an undirected graph and says nothing by itself about global workspace function, embodiment, or agentic world modeling. This difference is important when considering expander-like or error-correcting architectures: dense graph connectivity may support robust communication, but IWMT explicitly argues that such structure is not by itself sufficient for consciousness without cybernetic and embodied grounding [33]. The present results are compatible with that caution, since highly uniform redundant graphs are not especially informative under our metric.

**Criticality.** IWMT and related perspectives also suggest that consciousness-relevant dynamics may live near critical regimes in which coordination and differentiation coexist. The present transitional example is at least qualitatively consistent with that possibility: the most interesting regime for $\Phi_{\mathrm{spectral}}$ appears to lie between disorder and total synchrony, not at either extreme [33,38]. However, we did not directly measure self-organized criticality, branching statistics, or phase-transition markers, so this connection remains conjectural.

**Consciousness thresholds and PCI.** The current study also does not justify any non-arbitrary threshold of $\Phi_{\mathrm{spectral}}$ for consciousness. Any such threshold would need to be calibrated empirically against benchmark conscious and unconscious biological systems, and even then its extension to artificial systems would remain uncertain because the mapping from functional integration to phenomenal consciousness is unresolved. Relative to PCI, the contrast is sharp. PCI perturbs the cortex and measures the compressibility of the distributed response, and it has been validated as a discriminator of conscious level across wakefulness, sleep, anesthesia, and some disorders of consciousness [24]. By contrast, $\Phi_{\mathrm{spectral}}$ is passive and currently unvalidated clinically. It may prove useful as a complement where perturbation is impossible or continuous monitoring is desirable, but it is not a replacement for PCI.

**Validation still needed.** Several empirical steps now follow directly from the current analysis: comparisons against exact IIT calculations on tractable small systems; stationary coupling sweeps for Kuramoto-like models; multiple random seeds and sensitivity analyses over binning and window length; and perturbational tests to determine whether the statistic responds to causal interventions or only to observational redundancy. Until such validation is performed, claims about causal irreducibility should remain cautious.

## 6. Implications and Future Work

The value of $\Phi_{\mathrm{spectral}}$ lies in its computational tractability. Because it is defined directly on observed multivariate time series, it can be computed in settings where full causal reconstruction is impossible. This makes it potentially useful for monitoring large distributed systems in neuroscience, swarm robotics, networked control, and multi-agent AI. In such settings, a fast observer-relative measure of whether dependencies are spreading across a system may already be useful, even when a full intrinsic account is unavailable.

### 6.1. Applications to Intelligent Systems

In applied settings, $\Phi_{\mathrm{spectral}}$ could be used as a screening statistic for changes in modularity, redundancy, or distributed coordination. Examples include monitoring training dynamics in multi-agent learning, tracking loss of differentiation in synchronized sensor networks, or detecting compressive collapse in structured recurrent systems. These applications do not require identifying the statistic with consciousness or with exact IIT; they require only that the statistic behave reliably as an observer-level indicator of changing integration.

### 6.2. Consciousness-Related Use and Empirical Calibration

If $\Phi_{\mathrm{spectral}}$ is used in consciousness-related contexts, it should be used only as a tentative marker and not as a necessary or sufficient criterion. The current paper provides no principled threshold for consciousness, and no such threshold should be inferred from the figures presented here. A responsible program would first calibrate the statistic against biological benchmark states, compare it explicitly with perturbational measures such as PCI, and then assess how far any resulting discriminative range can legitimately be generalized to non-biological systems.

This restraint also helps with the exclusion issue mentioned above. Because $\Phi_{\mathrm{spectral}}$ can be computed for nested subsystems, it is naturally suited to multiscale exploratory analysis. That is a methodological advantage for complex systems research, but it also means that the measure is not by itself a theory of where consciousness “really” resides. Such questions require additional theoretical commitments that go well beyond the present statistic.

### 6.3. Directions for Future Research

Several natural extensions follow from the present work:

- **Small-system benchmarks.** Compare $\Phi_{\mathrm{spectral}}$ directly with exact or accepted IIT-style calculations on systems small enough for tractable computation.
- **Stationary parameter sweeps.** Replace time-varying coupling schedules with stationary sweeps over fixed coupling values, and report multiple seeds and confidence intervals.
- **Estimator sensitivity.** Evaluate robustness to bin count, window size, stride, and alternative estimators of pairwise dependence.
- **Beyond pairwise dependence.** Incorporate higher-order information, synergy, and redundancy to test whether multivariate structure changes the ordering of regimes.
- **Directed variants.** Explore transfer entropy, Granger-causal, or intervention-based analogs that better capture directionality and controllability.
- **Topology and attractors.** For CTLNs and related graph-based systems, combine spectral integration with attractor analysis, fixed-point support structure, and graph motifs.

Taken together, these directions point toward a broader research program: not the replacement of IIT by a single easy statistic, but the development of a family of scalable observer-level measures whose strengths and limitations are clearly characterized.

## 7. Conclusions

This paper introduces $\Phi_{\mathrm{spectral}}$, a normalized spectral cut statistic on pairwise mutual information graphs. The measure is inspired by IIT’s concern with irreducibility but should be interpreted more modestly as a tractable measure of observer-relative second-order integration. Across four exploratory simulation regimes, it distinguishes noisy independence, near-uniform redundancy, and topology-driven compression, while also revealing an important limitation: when the mutual information graph becomes nearly uniform, the Fiedler partition becomes degenerate and the statistic loses specificity.

The present contribution is therefore twofold. First, it provides a scalable way to track how pairwise informational dependencies distribute across large observed systems. Second, it clarifies the methodological conditions under which that statistic is informative and where stronger validation is still needed. With explicit small-system benchmarks, stationary coupling studies, and perturbational tests, $\Phi_{\mathrm{spectral}}$ could become a useful component of the broader weak-IIT and complex-systems toolbox.

## Data Availability

The raw data supporting the conclusions of this article will be made available by the authors on request.
